# Thymic Adenocarcinoma With Metastasis to the Left Orbit: A Case Report

**DOI:** 10.7759/cureus.56139

**Published:** 2024-03-14

**Authors:** Renata Quevedo, Sebastian Garcia, John C Cravero, Andrew Horton, Blaine Berger, Roberto I Aguirre

**Affiliations:** 1 Internal Medicine, Tecnologico de Monterrey, Monterrey, MEX; 2 Internal Medicine, Baylor Scott & White Medical Center - Temple, Temple, USA; 3 Pathology, Baylor Scott & White Medical Center - Temple, Temple, USA

**Keywords:** thymic epithelial tumors, thymic carcinoma, orbit metastasis, mediastinum, enteric-type thymic adenocarcinoma

## Abstract

We present the case of a 57-year-old female who initially presented with a chief complaint of left-sided orbital headaches and associated left eyelid swelling. Initial imaging work-up with CT head/orbit revealed soft tissue enhancement of the left orbital roof, concerning for neoplastic process (primary lymphoma versus extracranial primary tumor versus metastatic tumor). Further imaging studies with CT chest/abdomen/pelvis revealed an anterior mediastinal mass, concerning for possible thymoma versus lymphoma. The patient underwent further consultation with the Hematology/Oncology and Ophthalmology Departments, which recommended definitive biopsies from both sites, which showed matching histologic findings of moderately differentiated enteric-type adenocarcinoma with positive staining for CDX2, an intestinal marker. Thymic carcinomas are rare cancers that account for approximately 0.06% of all malignancies and require a high degree of clinical suspicion. Extrathoracic metastases from thymic carcinomas, especially to the orbit, is a rare occurrence and the exact incidence of this phenomenon is unknown. This case represents the diagnostic challenges associated with a rare cancer type and underscores the importance of a multidisciplinary approach to patient care.

## Introduction

The thymus is a small gland located behind the sternum that holds an important role in our immune system regarding the maturation and differentiation of T lymphocytes; under normal circumstances, it gradually undergoes involution and is virtually undetectable in post pubertal humans [1]. Thymic carcinomas (TCs), in general, are rare cancers that account for approximately 0.06% of all malignancies [2]. Primary thymic adenocarcinomas (TAdCas), which are a subtype of thymic epithelial tumors (TETs), are exceptionally rare and thymic enteric-type adenocarcinoma (AdCa) has recently been proposed as a distinct pathological entity [3]. Its diagnosis may have considerable difficulty due to its lack of specific diagnostic features, requiring clinical, radiological and histopathological correlations [4]. 

## Case presentation

**Figure 1:**
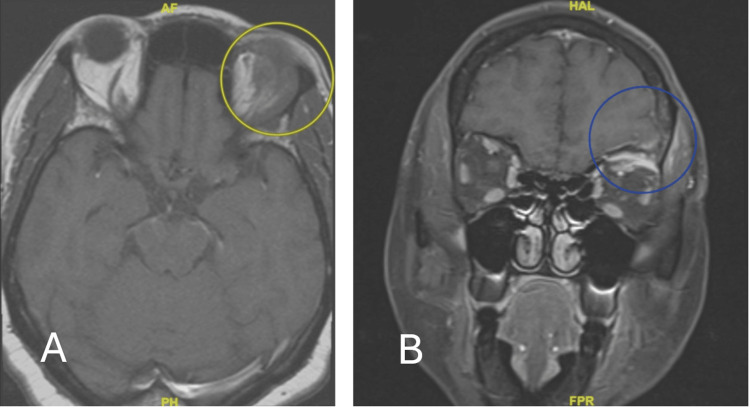
MRI orbits without contrast (March 2023) Abnormal appearance of the superior lateral extraconal soft tissues of the left orbit as denoted by the yellow circle in image A (axial view) and the blue circle in image B (coronal view). Mild superior periorbital soft tissue swelling with mild involvement of the left lacrimal gland is also present. Extraocular muscles, globe, and intraconal soft tissues are normal in appearance.

The patient was a 57-year-old female originally from Cameroon who presented to their primary care physician in January 2023 and complained of left eye and orbit swelling, concerning for pre-septal cellulitis. However, her swelling continued to persist, and she gradually began to suffer from left-sided headaches. She was evaluated by the Outpatient Ophthalmology Department in March 2023 and was empirically treated for giant cell arteritis (GCA) with high-dose prednisone. Initial imaging studies at that time revealed left orbital roof soft tissue enhancement, adjacent frontal bone enhancement, and some involvement of the lacrimal gland (Figure 1).

Given these findings, the patient underwent a left anterior orbitotomy with biopsy, where histological examination revealed normal lacrimal gland tissue infiltrated by chronic inflammation, thus excluding lymphoma. By May 2023, she presented to the Emergency Department for persistent headaches, restlessness, nausea, and intermittent diplopia associated with pronounced left eye swelling. She denied any history of trauma, vision alterations, insect bites, dysphagia, constitutional symptoms, or ear symptoms. Vital signs were within normal limits, and laboratory results, except for an elevated erythrocyte sedimentation rate (ESR) of 38, were unremarkable. She was hospitalized for further evaluation.

**Figure 2:**
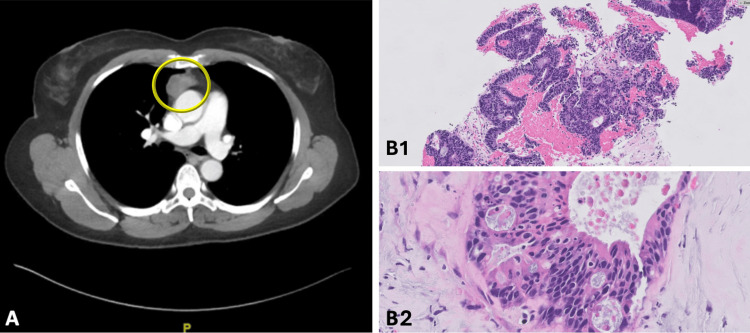
CT chest with contrast and biopsy of anterior mediastinal mass (May 2023) A.  Soft tissue mass within the anterior mediastinum as denoted by the yellow circle measuring 3.7 x 2.1 x 4.3 cm concerning for lymphoma versus thymoma. B1 and B2. Moderately differentiated enteric-type adenocarcinoma: features consistent with glandular structure formation, pseudostratified columnar cells with atypical nuclei, hyperchromasia, and a desmoplastic stroma with inflammatory infiltrate. Pathology specimens stained with H&E. B1 magnification 100x and B2  magnification 400x.

A physical examination revealed erythema and edema of the left superior eyelid with minimal conjunctival injection. Imaging, including CT head and CT orbits with contrast, exhibited extraconal soft tissue enhancement in the left orbital roof, adjacent frontal bone sclerosis, and soft tissue enhancement proximal to the right temporalis muscle. These findings raised concern for an extracranial meningioma versus lymphoma. During her course, she experienced worsening edema of the left periorbital region, ptosis, and the emergence of a left bulging soft tissue swelling in the frontal lobe directly above the affected eye. A CT chest with contrast and a CT abdomen and pelvis were performed to assess for other sites of disease, which revealed a mass within the anterior mediastinum, concerning for lymphoma versus thymoma (Figure 2).

**Figure 3:**
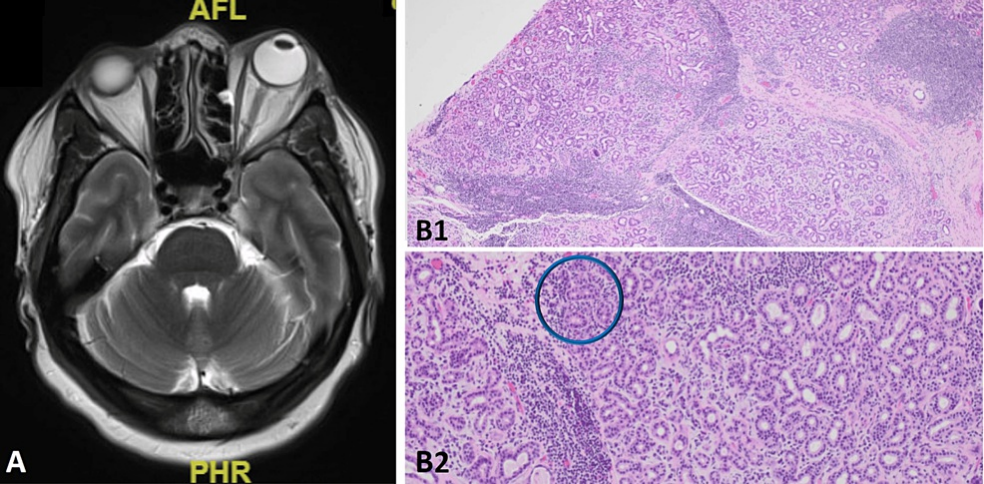
MRI orbits without contrast and left superior orbital mass biopsy (May 2023) A. Progressive since March, an infiltrative mass involving the left superolateral pre- and post-septal orbits with predominant involvement of the extraconal soft tissues but new interval extension up to and involving the left superior oblique muscle. B1 and B2. Focal moderately to poorly differentiated adenocarcinoma with enteric features. An area of high-grade adenocarcinoma is identified within the blue circle. This tumor appears morphologically similar to the patient's adenocarcinoma within the mediastinum, consistent with metastasis. Pathology specimens stained with H&E. B1 magnification 20x and B2 magnification 100x.

Ophthalmology and hematology/oncology consultations were conducted. While awaiting a biopsy of the anterior mediastinal mass, an ophthalmologic examination revealed left adduction, infraduction, and supraduction deficits suggestive of a partial left oculomotor (third) cranial nerve (CN III) palsy. Further MRI of the brain and orbits without contrast was recommended to evaluate extraocular muscle extension of the orbital mass; the findings were most consistent with progressive left orbital lymphoma (Figure 3), which prompted evaluation with a left orbital mass biopsy.

The final biopsy results of the anterior mediastinal mass demonstrated cells that resembled colorectal adenocarcinoma and stained positive for CDX2, CK20, and SATB2 and negative for CK7 and TTF1, supporting a diagnosis of moderately differentiated enteric-type AdCa (Figure 2). In the absence of other imaging findings and a prior colonoscopy from August 2022 without hyperplastic or adenomatous findings, it was felt that the mediastinal mass was likely thymic in origin. The patient underwent a left anterior orbitotomy with a biopsy of the left superior orbital mass with a transcutaneous approach via lid crease incision. The subsequent biopsy results of the orbital mass demonstrated the same histology as the anterior mediastinal mass (Figure 3), further supporting a diagnosis of metastatic thymic carcinoma. The patient was ultimately discharged with further outpatient follow-up with the Hematology/Oncology Departments, which recommended treatment with oxaliplatin/5-fluorouracil/leucovorin (FOLFOX) regimen due to the histologic similarity between the thymic tumor and AdCa of the colon. Based on the rarity of her diagnosis and clinical presentation, the patient was ultimately referred to MD Anderson Cancer Center, Texas, United States, for further expert evaluation. 

## Discussion

Thymic carcinomas (TCs) account for about 20% of all TETs [5]. Moser et al. introduced the concept of primary thymic AdCa of the enteric type as a distinct subtype of TC [6]. In 2021, WHO reclassified mucinous AdCas into two categories, either enteric-type AdCas if the expression of one or more of the enteric markers CK20, CDX2, or MUC2 were present or into the AdCas not otherwise specified (NOS) category [7,8]. Thymic enteric-type AdCa is exceedingly rare; the number of reported cases as of 2021 was only 16, and its clinical features and treatment options are still not well-defined [9,10]. While the exact incidence of extrathoracic thymic carcinoma metastasis is unknown, various sites in the liver, lungs, adrenal glands, kidney, and brain have been reported [2]. Of note, thymic carcinoma of varying histologic types with metastases to the orbit have also been described but are exceedingly rare [11,12]. Some patients present with nonspecific symptoms such as chest pain, headache, cough, or shortness of breath, while others remain asymptomatic [13]. Based on the complexity and rarity of these findings, a primary enteric-type TAdCa necessitates a high level of awareness and careful clinicopathological correlation for accurate diagnosis. 

Our patient had an unusual metastatic cancer presentation within the orbital region and was treated empirically with high-dose prednisone for suspected GCA, which may have temporarily masked the cancer-related symptoms, adding complexity to the diagnostic process. However, the delay in the diagnosis allowed the cancer to progress, resulting in significant symptoms such as diplopia, pronounced eye swelling, and left-eye muscle deficits.

Regarding treatment, surgery is considered the main treatment for patients when feasible. Unfortunately, only a fraction of patients, typically ranging from 46% to 68%, are suitable candidates due to the advanced stage at which many of these tumors are diagnosed [14]. Chemotherapy and radiation therapy are used to treat locally advanced disease and systemic therapy alone is indicated for metastatic TETs [5]. For advanced, unresectable (Stage III or Stage IV) disease, treatment with carboplatin/paclitaxel is the preferred first-line systemic treatment [15]. FOLFOX and capecitabine/oxaliplatin (XELOX) regimens, originally designed for colorectal cancer, have shown promise as alternative options in enteric-type AdCa due to shared histological and molecular features [15]. The use of immunotherapy drugs like pembrolizumab, an anti-PD1 monoclonal antibody, and antineoplastic agents, such as sunitinib, showcase the potential for improved patient outcomes and underscores the importance of tailoring treatments to the specific characteristics of this tumor [9,11].

Thymic carcinomas have a reported five-year survivability rate of only around 33.8% to 35.6%, emphasizing that the approach and specific treatment plan for a metastatic TAdCa depends on many factors, such as the extent of the cancer, the patient’s health, and overall clinical presentation [14].

## Conclusions

This case shows the complex journey of a patient with a rare presentation of Stage IV TAdCa. The exceptionality of her diagnosis, especially with orbital metastasis, poses challenges for the medical community, emphasizing the value of an individualized, multidisciplinary approach. Increasing awareness of this type of cancer and emphasizing the need to biopsy primary and metastatic sites can prevent diagnostic delay and ensure that the right treatment is initiated. 
